# Tsetse Flies (*Glossina*) as Vectors of Human African Trypanosomiasis: A Review

**DOI:** 10.1155/2016/6201350

**Published:** 2016-02-29

**Authors:** Florence Njeri Wamwiri, Robert Emojong Changasi

**Affiliations:** Kenya Agricultural and Livestock Research Organisation, Biotechnology Research Institute, P.O. Box 362, Muguga 00902, Kenya

## Abstract

Human African Trypanosomiasis (HAT) transmitted by the tsetse fly continues to be a public health issue, despite more than a century of research. There are two types of the disease, the chronic* gambiense* and the acute* rhodesiense*-HAT. Fly abundance and distribution have been affected by changes in land-use patterns and climate. However, disease transmission still continues. Here, we review some aspects of HAT ecoepidemiology in the context of altered infestation patterns and maintenance of the transmission cycle as well as emerging options in disease and vector control.

## 1. Introduction

African trypanosomiasis is one of a diverse range of neglected tropical diseases. The tsetse fly,* Glossina sp*. is the main vector for trypanosomes, the parasites that cause trypanosomiasis. This disease affects both humans and livestock. In humans, the disease is known as sleeping sickness or Human African Trypanosomiasis (HAT) while, in livestock, it is referred to as nagana or African Animal Trypanosomiasis (AAT). AAT is widespread across most of the 38 countries of sub-Saharan Africa that are considered endemic for tsetse flies and the disease [1] and is considered to be a major factor limiting agricultural production [2]. On the other hand, HAT occurs as a highly focal disease [3]. Both the disease and its vector have been the focus of intense scientific interest since David Bruce first described the link between tsetse flies and HAT [4]. In spite of this, trypanosomosis continues to be a constraint to livestock-based rural livelihoods and a potentially fatal human disease [5].

## 2. Epidemiology of Sleeping Sickness

There are two distinct forms of sleeping sickness, with differences in aetiology, epidemiology, clinical manifestation, and treatment regimes. The chronic anthroponotic form which is caused by* Trypanosoma brucei gambiense* (*g*HAT) occurs in 24 countries in west and central Africa and accounts for about 98% of reported cases (WHO Technical Report 2012). The Democratic Republic of Congo (DRC) continues to report the highest number of* g*HAT cases, contributing up to 84% of endemic cases reported in 2012 [6]. The acute zoonotic form of the disease caused by* T. b. rhodesiense* is termed as* rhodesiense*-HAT (*r*HAT) and is found in 13 countries in eastern and southern Africa. Less than 2% of reported HAT cases are due to* T. b. rhodesiense* [7]. Uganda has the distinction of being the only country with both* r*HAT and* g*HAT; however, the incidence foci of two diseases are spatially distinct and no convergence of the two disease zones has been observed yet [8].

### 2.1. Disease Transmission Cycle

The transmission cycle of* gambiense*-HAT (*g*HAT) is most commonly considered to be human-fly-human. It is thought that, in the presence of the vector, the long duration of* g*HAT infection in humans is sufficient to maintain the transmission cycle. This forms the basis of the traditional screen-and-treat approach to* g*HAT control. A possible animal reservoir has been suggested in the epidemiology of* g*HAT, but its contribution to disease transmission remains unclear. Indeed, surveys conducted in* g*HAT foci indicate the presence of no or very few* T. b. gambiense* infections in livestock or wild animals [9, 10]. Additionally, it has been possible to locally eliminate* T. b. gambiense* transmission by treatment of the human reservoir alone, without recourse to animal targeted interventions [11]. However, the involvement of animals in this cycle cannot be completely ruled out. It has been suggested that sustainable control of AAT is an indispensable approach towards achieving* g*HAT elimination in west and central Africa [12].

On the other hand, the transmission of* r*HAT relies on the presence of vertebrate reservoirs comprising both domestic and wild animals [13] and the cycle is typically animal-tsetse-animal/human. However, during epidemics when the number of infected persons is relatively high, the transmission cycle may follow the route human-tsetse-human [14]. In eastern and southern Africa, numerous wildlife species living in conservation areas serve to maintain the disease reservoir. As a result, some of the people infected do so in or around the game parks or reserves [15]. In the period 1990–2007, 49 nonendemic cases were documented; principally tourists who were presumably exposed to tsetse bites in game parks in Kenya, Malawi, Tanzania, Uganda, Zambia, and Zimbabwe (reviewed by [16]). Although wild animals are the key reservoirs in these game parks, investigations reveal low infection rates and low parasitaemia levels with human-infective trypanosomes [17]. For instance, a recent study conducted within Zambia's Luangwa Valley found a paltry 0.5% (*n* = 418)* T. b. rhodesiense* infection prevalence in surveyed wildlife [18]. Livestock in low endemicity foci exhibit a correspondingly low prevalence of* T. b. rhodesiense*, but this can be enough to cause a flare-up of the disease. The emergence of HAT in northern Uganda has been linked to the introduction of infected cattle from endemic southern areas in a restocking programme [19] and highlights the important role of veterinary policy in mitigating the effects of zoonotic disease [20].

### 2.2. Trends in Numbers of Reported Cases

The incidence of sleeping sickness has been declining over the years, falling from circa 26,000 cases reported in 2000 to less than 8,000 cases reported in 2012. Specifically, the number of* g*HAT and* r*HAT cases reported to WHO in this period decreased by 75.9% and 87.9%, respectively [14]. This decrease is attributed to improved case detection and treatment and vector management [21]. Despite this decreased incidence, it is estimated that up to 70 million people distributed over 1.5 million km^2^ remain at risk of contracting the disease [22].

### 2.3. Effect of Changes in Land-Use and Climate on Disease Risk and Incidence

Rising population levels in many parts of sub-Saharan Africa have caused increased land pressure, pushing more people into tsetse infested marginal areas. This immigration has led to one of two outcomes: (i) elimination of tsetse habitat, hence disappearance of tsetse flies and apparent elimination of the disease [23], or (ii) increased human-fly contact, leading to increased risk of contracting trypanosomiasis [24]. Simulation models have suggested that population growth will cause a decline of savannah and forest tsetse, with possible extinction in eastern and southern Africa [25]. In these regions, tsetse populations have been confined to discrete habitats, with a high abundance in and around wildlife conservation areas such as game parks and reserves. Such conservation areas provide suitable conditions for tsetse survival and function as breeding sites. With increased human encroachment into protected areas, there is bound to be an increased disease risk [26], at least in the initial period of settlement.

Transmission of vector-borne diseases, including trypanosomiasis, is influenced by the environment, and any changes in that environment may affect the disease, hence their impact on health and the economy [27]. In traditional HAT foci, environmental and biological conditions are ideal for the coexistence and interaction of the vector, the host, and the parasites, thus permitting disease transmission to take place. Factors that affect the resting sites for adult tsetse flies, such as long-term changes in rainfall and temperature, can have a significant effect on the epidemiology and transmission of trypanosomiasis [28]. In both Burkina Faso and Mali decreased rainfall and increased human density have been implicated in the contraction of previously documented tsetse habitat limits [29]. Further, fragmentation of the tsetse habitat has important effects on fly population dynamics and has been shown to reduce tsetse apparent densities [18].

Landscape features and livestock and human mobility are all important predictors of HAT incidence as they influence fly presence, density, and dispersal [30]. Social, cultural, and economic factors also affect outcomes in disease incidence. A comparative analysis of socioeconomic and cultural determinants of HAT in four adjacent foci on the boundary of Kenya-Uganda border concluded that knowledge of tsetse and its control, culture, farming practices, and demographic and socioeconomic variables explained occurrence of HAT better than landscape features [31]. These sociocultural practices may also be used to explain the phenomenon of sleeping sickness patients presenting in urban health centers, particularly in the* g*HAT foci of central Africa.* Palpalis* group tsetse, of which the* G. fuscipes* subspecies are estimated to be responsible for about 90% of all HAT cases [32] inhabit fairly conserved riparian environments [31, 33]. The flies of this group are able to adapt to and easily colonize peridomestic habitats [25], including suburban areas surrounding cities, for example, Kinshasa, Libreville, Bonon, and Bangui [34, 35]. These foci have been termed as “rural foci with urban manifestation” whereby infection does not typically occur within the city limits, but people get infected in the course of their forays into the tsetse-infested periphery of the city [36].

## 3. Tsetse Flies as Vectors of Human-Infective Trypanosomes

Tsetse flies can be grouped into three main subgroups depending on the environment they inhabit: thus, riverine (*palpalis*), savannah (*morsitans*), or forest-dwelling tsetse (*fusca*). All tsetse species are capable of transmitting human-infective trypanosomes. However, the major species involved in HAT transmission are the* palpalis* group tsetse, specifically* G. palpalis spp* and* G. fuscipes spp*. Sleeping sickness occurs in geographically delineated zones referred to as “foci” [37]. Such foci are often infested by sympatric species, whereby one species is the predominant one [38, 39]. Flies pick up bloodstream parasites from their hosts: livestock, wildlife, and humans. Vectorial capacity describes the innate ability of a specific fly species to acquire, mature, and transmit trypanosomes. Different tsetse species coinfesting the same habitat often have varying vectorial capacities for human-infective trypanosomes [40, 41]. For this reason, it is important to determine the infection prevalence in sympatric tsetse species so as to identify which species are key in disease transmission. Such data can then be used to inform decisions on control interventions. In addition, infection prevalence data helps scientists to better understand transmission dynamics and detect spatiotemporal trends, both of which have important implications for disease control [42]. However, in nature, the prevalence of human-infective trypanosomes in tsetse flies, as detected by parasitological methods (dissection and microscopy), is often very low [42], even in active foci [43, 44]. The classical dissection/microscopy technique [45] though labour-intensive may be the only tool available to determine infection rates in the field. Using dissection,* T. brucei* infections are indicated by the presence of trypanosomes in the salivary glands. This procedure however has disadvantages in that it requires skilled technicians and has a low diagnostic sensitivity [42, 46, 47]. In many cases, dissection results do not vary much in epidemic or endemic situations and is often less than 1%, despite available evidence of active infections in animals or humans [42, 48–50]. The PCR technique is frequently applied to detect parasite DNA in disease vectors [51, 52]. However, the presence of parasite DNA does not indicate the presence of a mature, transmissible infection and therefore is not a direct indicator of risk [42]. Often, PCR gives a misleading overestimation of fly infection as compared to dissection results. This is because PCR detects trypanosome DNA and will not differentiate between an active transmissible infection in the fly and a recent infective feed. This therefore necessitates the development and use of novel methods to corelate prevalence with disease risk.

## 4. Emerging Options in Disease and Vector Control

Despite considerable investments towards its control and/or eradication, tsetse and trypanosomiasis still remain a major public health issue. The control of sleeping sickness hinges on two key aspects: disease control and vector control. Recent and ongoing improvements to these two aspects are contributing to attainment of the WHO target for disease elimination.

### 4.1. Integration of HAT Diagnosis and Treatment into National Primary Health Care Systems

The resurgence of sleeping sickness in countries such as Sudan, Angola, and the DRC has been attributed to political and civil unrest which has resulted in mass migration of populations into risk situations and the breakdown of traditional government support and disease control systems [53]. In many of these countries, disease surveillance and control activities are highly dependent on foreign aid, including nongovernmental aid agencies [54, 55]. The reduction or/and cessation of foreign aid can affect control activities, leading to flare-ups of the disease. In the case of DRC, a dramatic upsurge in number of cases (up to 25,000 cases annually) was experienced when Belgian bilateral aid funding disease surveillance and treatment activities was terminated in 1990. The trend was reversed with the resumption of bilateral aid in 1998, and the subsequent continuation of large scale screening activities and treatment programmes [56]. To reduce dependency on foreign aid for HAT control, endemic countries are being encouraged and supported to take up ownership of the control process [57]. Towards this, efforts have been made towards integration of disease diagnosis and treatment activities at government primary health care centers [58].

### 4.2. Improved Diagnostic Tools

Substantial progress has also been made towards the development and routine application of improved diagnostic tools in endemic countries. These include novel and/improved techniques, for example, those incorporating the use of light-emitting diode fluorescence microscopy [59], Loop-Mediated Isothermal Amplification (LAMP) technique [60], and individual Rapid Diagnostic Tests (RDTs) [61] are currently undergoing evaluation towards routine usage as point-of-care tests [62]. In addition, new algorithms are being developed to shorten time-to-treatment for* g*HAT [63] which effectively reduces the possibility of onward transmission. Another development towards improved HAT control has been compilation of disease distribution maps for HAT, taking advantage of the focal nature of the disease to compile comprehensive village-level maps of HAT distribution as an essential tool for disease control, research, and advocacy [7]. The HAT Atlas provides a valuable contribution towards informed decision-making for planning and monitoring of control activities and assessment of epidemiological trends as well as research activities.

### 4.3. Inclusion of Vector Management Strategies as a Key Component of* g*HAT Control

It has long been widely accepted that tsetse control plays a central role in controlling the zoonotic* r*HAT [64]. However, epidemiologists now concur that vector control is required in the management of* g*HAT as well [44, 65]. Indeed, the implementation of vector control strategies along with medical interventions (screen and treat) in several* g*HAT foci, including Mandoul (Chad), northwestern Uganda, and Boffa (Guinea), has significantly reduced the incidence of new cases [66, 67]. The necessity of vector control is also supported by the chronic nature of* g*HAT infection, with one case reportedly presenting as long as 29 years after the initial infection [68]. In the presence of the vector, such an asymptomatic carrier may have an important potential role in disease transmission. There have been considerable advances made in the search for efficient and cost-effective control tools against riverine tsetse involved in the transmission of* g*HAT [21]. This search has culminated in the development of so-called “tiny targets” [69]. These insecticide-treated targets are much smaller (25 cm × 50 cm) than the traditional 1 × 1 m targets. Despite their size, tiny targets have proved to be quite effective for the control of riverine tsetse, specifically* G. fuscipes* spp. and* G. palpalis* spp. [70, 71]. In addition, they cost much less due to their smaller size (hence reduced costs for impregnation and materials). In addition, due to their light weight, they can be easily deployed by foot, or using bicycles and motorcycles [72]. Further, tiny targets when mounted on pirogues moving along a river have proved to be effective in reducing tsetse density [73]. The development of such novel control tools, as well as standardization of existing models, will lead to identification of cost-effective devices for tsetse management [70, 74, 75].

### 4.4. Adoption of the “One Health” Concept in HAT Control

Increasingly, integrated strategies that use interdisciplinary study and action to address control of both HAT and AAT simultaneously are being promoted. This approach of controlling pests of veterinary importance that transmit zoonotic agents is an example of the “One Health” concept, where a single vector control technique mitigates the risk for transmission of two diseases. Indeed, the WHO specifically recommends that surveillance and control of* r*HAT should be coordinated with veterinary services in a “One Health” approach [76]. Today, this strategy has been widely applied in both* g*HAT and* r*HAT foci to decrease tsetse density, thereby reducing human-tsetse contact and proving that simultaneous control of both AAT and HAT has a bigger impact on disease incidence [77–79].

### 4.5. Vector Control in Protected Areas

In eastern and southern Africa, the distribution of tsetse flies is increasingly confined to protected areas such as game parks and reserves. These parks, due to their suitable vegetative cover and array of available host species, act as tsetse breeding sites and a large number of current* rhodesiense* foci are allied to game parks. This situation has resulted in increased risk and incidence of* r*HAT infection among tourists and game park staff [80], forcing some countries to implement tsetse control in game parks. In collaboration with tsetse control experts, wildlife authorities have instituted measures to reduce human-fly contact including aerial spraying, installation of impregnated traps, and targets/screens and spraying of vehicles on park exit [81–83]. Such interventions may be successful if they are sustained for prolonged periods of time and should therefore be promoted.

### 4.6. Future Control Options Using a Paratransgenic Approach

Another development towards vector management in HAT control comes from the field of genetic modification. Arthropod research has revealed the presence of symbionts involved in the suppression of pathogenic organisms [84–86] and which can be manipulated to express foreign proteins designed to block pathogen transmission [87]. This strategy, known as paratransgenesis, has been developed and proposed to combat different insect-borne animal and human diseases [85, 88–90]. The paratransgenic approach has been proposed as a strategy for inhibiting trypanosome survival, development, and maturation in tsetse and therefore interference with transmission of African sleeping sickness [91]. The tsetse symbiont,* S. glossinidius*, has been shown to influence vector competence, at least in some fly species [92–96]. For this reason, this bacterium is considered as a potential* in vivo* drug delivery vehicle to control trypanosome development in the fly [97]. The availability of* in vitro* cultures of* S. glossinidius* has enabled development of genetic transformation systems that introduce and express foreign products in* Sodalis* and subsequently into host insects [85]. Along this line, Belgian researchers have successfully genetically modified* Sodalis* to express antitrypanosomal genes that specifically target bloodstream parasites. This is a proof-of-concept that indeed the* Sodalis* bacterium is able to express and release a sufficient amount of active, functional, parasite-targeting compound [97]. This discovery provides a promising avenue in the fight against tsetse-transmitted trypanosomiasis.

## 5. Future Prospects

After more than 100 years of tsetse and trypanosomiasis research, the prognosis of the disease remains ambiguous. There is a prominent idea that vector eradication may be impossible to achieve, even with sustained integrated approaches but these may be enough to maintain high levels of suppression [98]. In addition, it is thought that elimination of* T. brucei rhodesiense* is unlikely due to its extensive zoonotic distribution. Given that, future research towards disease control should focus on improvement of vector control methods, cost-effective disease surveillance, and early case detection and treatment [78]. However, some outlooks are generally more positive. The WHO, by including HAT in its roadmap for “Eradication, Elimination, and Control of Neglected Tropical Diseases” has set a target to eliminate HAT as a public health problem by 2020, when fewer than one new case/10,000 inhabitants in at least 90% of endemic foci is expected [76]. It is argued that transfer of all the accumulated scientific knowledge on tsetse and HAT from the bench to the field will lead to effective diagnosis, treatment and vector control interventions [99]. In particular, the elimination of* g*HAT is considered feasible because of “the epidemiological vulnerability of the disease, the current state of control, the availability of strategies and tools, and international commitment and political will” [14].
